# Patellar cartilage increase following ACL reconstruction with and without meniscal pathology: a two-year prospective MRI morphological study

**DOI:** 10.1186/s12891-021-04794-5

**Published:** 2021-10-28

**Authors:** Xinyang Wang, Kim L. Bennell, Yuanyuan Wang, Karine Fortin, David J. Saxby, Bryce A. Killen, Tim V. Wrigley, Flavia M. Cicuttini, Ans Van Ginckel, David G. Lloyd, Julian A. Feller, Christopher J. Vertullo, Tim Whitehead, Price Gallie, Adam L. Bryant

**Affiliations:** 1 Centre for Health, Exercise and Sports Medicine, Department of Physiotherapy, School of Health Sciences, The University of Melbourne, 161 Barry Street, Carlton, Victoria 3010 Australia; 2 School of Public Health & Preventive Medicine, Monash University, Melbourne, Victoria Australia; 3 Faculty of Arts, Monash University, Melbourne, Victoria Australia; 4 School of Allied Health Sciences, Griffith University, Gold Coast, Australia; 5 Griffith University Centre for Biomedical and Rehabilitation Engineering (GCORE), Menzies Health Institute Queensland, Gold Coast, Australia; 6 Human Movement Biomechanics Research Group, KU Leuven, Leuven, Belgium; 7 Department of Rehabilitation Sciences, Ghent University, Ghent, Belgium; 8 OrthoSport Victoria, Melbourne, Australia; 9 College of Science, Health and Engineering, La Trobe University, Melbourne, Australia; 10 Knee Research Australia, Gold Coast, Australia; 11 Coast Orthopaedics, Gold Coast, Australia

**Keywords:** Anterior cruciate ligament reconstruction, Post-traumatic osteoarthritis, Cartilage volume, Cartilage defects, Patellofemoral joint

## Abstract

**Background:**

Anterior cruciate ligament reconstruction (ACLR) together with concomitant meniscal injury are risk factors for the development of tibiofemoral (TF) osteoarthritis (OA), but the potential effect on the patellofemoral (PF) joint is unclear. The aim of this study was to: (i) investigate change in patellar cartilage morphology in individuals 2.5 to 4.5 years after ACLR with or without concomitant meniscal pathology and in healthy controls, and (ii) examine the association between baseline patellar cartilage defects and patellar cartilage volume change.

**Methods:**

Thirty two isolated ACLR participants, 25 ACLR participants with combined meniscal pathology and nine healthy controls underwent knee magnetic resonance imaging (MRI) with 2-year intervals (baseline = 2.5 years post-ACLR). Patellar cartilage volume and cartilage defects were assessed from MRI using validated methods.

**Results:**

Both ACLR groups showed patellar cartilage volume increased over 2 years (*p* < 0.05), and isolated ACLR group had greater annual percentage cartilage volume increase compared with controls (mean difference 3.6, 95% confidence interval (CI) 1.0, 6.3%, *p* = 0.008) and combined ACLR group (mean difference 2.2, 95% CI 0.2, 4.2%, *p* = 0.028). Patellar cartilage defects regressed in the isolated ACLR group over 2 years (*p* = 0.02; Z = − 2.33; *r* = 0.3). Baseline patellar cartilage defect score was positively associated with annual percentage cartilage volume increase (Regression coefficient B = 0.014; 95% CI 0.001, 0.027; *p* = 0.03) in the pooled ACLR participants.

**Conclusions:**

Hypertrophic response was evident in the patellar cartilage of ACLR participants with and without meniscal pathology. Surprisingly, the increase in patellar cartilage volume was more pronounced in those with isolated ACLR. Although cartilage defects stabilised in the majority of ACLR participants, the severity of patellar cartilage defects at baseline influenced the magnitude of the cartilage hypertrophic response over the subsequent ~ 2 years.

**Supplementary Information:**

The online version contains supplementary material available at 10.1186/s12891-021-04794-5.

## Background

Anterior cruciate ligament reconstruction (ACLR) is a common treatment following ACL injury. Whilst ACLR is typically effective in restoring anterior knee stability, a substantial portion of ACLR patients will develop early onset knee osteoarthritis (OA) – a painful and debilitating condition for which there is no known cure [1–4]. Traditionally, research has focused on tibiofemoral (TF) joint OA following ACLR; however, a high prevalence of OA in the patellofemoral (PF) joint following ACLR has been reported [5, 6] and which is characterised by knee symptoms such as pain and reduced function [5].

Meniscal injury frequently occurs at the time of the ACL injury and has been recognised as a risk factor for knee OA [1, 7, 8]. Biomechanical changes following meniscal injury or resection are thought to influence anterior-posterior laxity of the PF joint and internal-external rotation of the TF joint [9, 10]; thus, altered PF and TF joint biomechanics likely predispose the knee joint to OA development [11]. A previous study [5] reported that medial meniscal injury increased the risk of developing PF joint OA and medial TF joint OA at 5–10 years post-ACLR. A prospective evaluation of PF cartilage changes in ACLR knees with and without concomitant meniscal injury is warranted to understand PF joint OA cartilage pathophysiology.

Magnetic resonance imaging (MRI) is a non-invasive method to assess knee cartilage morphology with demonstrated sensitivity and reliability [12]. MRI studies have revealed alterations in cartilage morphology in the early years following ACL injury and ACLR [13, 14]. Morphological changes vary according to the knee compartment as both cartilage increases and decreases have been reported at 1–5 years post-surgery [15–18]. In particular, a longitudinal study demonstrated a general increase in TF joint cartilage thickness over a 5-year post-operative period [15], suggesting a alteration of cartilage homeostasis [15] prior to cartilage breakdown [15–17]. By contrast, a recent study reported a decreased in total PF joint cartilage thickness over 5 years following ACL injury [14]. Cartilage defects, a MRI-derived semi-quantitative cartilage morphology measurement, indicate early pathology following joint injury [18, 19]. Potter et al. [20] reported progressive worsening of patella cartilage defects following ACL injury over 11 years regardless of treatment (i.e., ACLR or conservative management). These findings demonstrated PF joint cartilage loss and degeneration following ACL injury/ACLR. In addition, the detrimental long-term effects of cartilage defects have been established in the TF joint. Whilst severe baseline cartilage defects were associated with cartilage loss in knee OA populations [21, 22], we found that mild baseline cartilage defects were associated with greater increases in cartilage volume in an ACLR cohort [23]. Overall, tibial cartilage volume increased after ACLR, and those with isolated ACLR exhibit greater increases in cartilage volume than those with ACLR combined with commitment meniscal pathology at the lateral tibia [23]. Thus, it is of interest to examine whether similar responses are apparent in the patellar cartilage.

The aims of this study were twofold. The first aim was to investigate the changes of patellar cartilage volume and cartilage defects from 2.5 to 4.5 years after ACLR in participants with: (i) isolated ACLR without meniscal pathology, (ii) ACLR combined with meniscal pathology, and (iii) healthy controls. It was hypothesised that: H_1_): ACLR knees would show increased patellar cartilage volume and increased cartilage defects score (progression), whilst control knees would show no change over 2-year; H_2_): knees with combined ACLR and meniscal pathology would show greater increase in cartilage volume and greater increase in cartilage defect scores compared with ACLR isolated knees. The second aim was to examine the association between baseline patellar cartilage defects scores and cartilage volume change in ACLR knees. It was hypothesised that: H_3_): higher cartilage defects at baseline would be associated with greater cartilage volume increase after 2 years.

## Methods

### Participants

One hundred participants who had undergone ACLR were recruited in Melbourne (Epworth Hospital Richmond) and Gold Coast (Pindara Hospital, Pacific Private Hospital, and John Flynn Hospital) Australia. Characteristics of these ACLR participants have been described in detail previously [23, 24]. Briefly, this relatively young cohort (i.e., 18–40 years) had undergone ACLR 2–3 years prior using the combined semitendinosus and gracilis autograft and none had evidence of tibiofemoral OA. Those with concomitant meniscal pathology (i.e., meniscal injury, repair or partial meniscectomy) were categorized to the combined ACLR and meniscal pathology group. A control group consisting of 30 healthy individuals without a history of knee injury and lower-limb surgery were also recruited.

ACLR surgery was arthroscopically assisted and has been previously described [23, 24]. Management of the meniscal injury (i.e., leave as is, repair, or partial meniscectomy) was determined by the surgeon based on the injury appearance at the time of surgery. Partial meniscectomy was performed for non-repairable meniscal injuries. No chondral surgery was performed as all lesions were not considered serious [International Cartilage Repair Society (ICRS) score grade < 3]. After being discharged from hospital, patients were encouraged to participate in weight bearing exercise on an as-tolerated basis without the use of braces or splints. The ACLR rehabilitation protocol targeted on rapid restoration of knee range of motion as well as quadriceps function.

### Anthropometry

Mass and height were measured, and then used to calculate BMI (kg/m^2^).

### Magnetic resonance imaging

The MRI protocol and assessment have been presented previously [24] and are briefly summarized below. The MRI scans were performed at baseline (i.e., 2–3 years post-ACLR) and at follow-up 2 years later using whole-body MRI units in Melbourne (3.0 T, Siemens Magenetom Verio, Erlangen, Germany) and the Gold Coast (1.5 T, GE Healthcare Signa, Wisconsin, USA). Knees were imaged using T_1_-weighted 3-dimensional gradient recall sequences in the sagittal plane [25].

### Patella cartilage volume and patella bone volume

Patella cartilage volume was measured based on the T_1_-weighted images using a previously published method [24, 25]. Images were transferred to Osiris v4.19 software (University Hospital of Geneva, Geneva, Switzerland) and cartilage was manually segmented by tracing the osteochondral interface and articular surface slice-by-slice. Cartilage volume (mm^3^) was determined by summing segmented areas and multiplying by slice thickness (1.5 mm). Baseline and follow-up cartilage volumes were measured in pairs for each participant by one trained assessor (XW) who was blinded to participant status. Intra-class correlation coefficients (ICC) for intra-rater and inter-rater reliability were 0.997 and 0.993, respectively [24]. Baseline patella bone volume was measured for statistical adjustment using the same method as for cartilage volume with ICCs above 0.98. The annual percentage change in patella cartilage volume was calculated as follows: (follow-up patella cartilage volume minus baseline patella cartilage volume) divided by (baseline patella cartilage volume multiplied by time period between scans). Positive values indicate increased cartilage volume over time.

### Cartilage defects

**Fig. 1:**
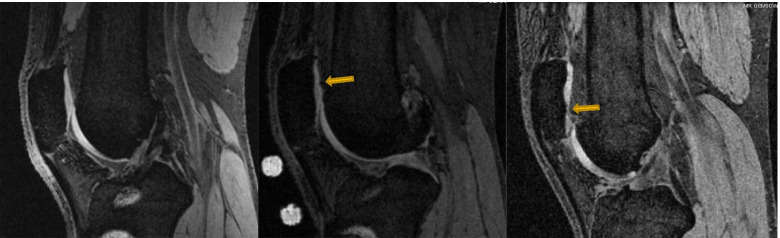
Patellar cartilage defect grade 0 (left), grade 2 (middle, arrow) and grade 3 (right, arrow)

Cartilage defects were assessed in T_1_-weighted images using the ICRS cartilage defect grade (score 0–4) as previously described [18, 24, 26]. The cartilage defects were graded as follows [18, 26]: grade 0, normal cartilage; grade 1, focal blistering and intra-cartilaginous low-signal intensity area with an intact surface and base; grade 2, irregularities on the surface or base thickness < 50%; grade 3, deep ulceration with loss of thickness > 50%; grade 4, full-thickness cartilage wear with exposure of subchondral bone (Fig. 1 Patellar cartilage defect grade 0 (left), grade 2 (middle, arrow) and grade 3 (right, arrow)). Intra-observer and inter-observer reliability, expressed as ICCs, were 0.94 and 0.93, respectively [24]. Cartilage defects were considered to have: 1) ‘progressed’ if defect grade increased (i.e., worsened), 2) ‘regressed’ if the defect grade decreased (i.e., improved), or 3) ‘stable’ if defect grade did not change over 2 years.

### Statistical analysis

Mean ± standard deviation was calculated for parametric variables, and changes over time were assessed using paired samples T-tests. Median ± interquartile range was calculated for non-parametric variables, and changes over time were assessed using Wilcoxon signed-rank tests. Participant characteristics were compared using independent samples T-tests or Chi-squared tests. Group-differences in annual percentage change in cartilage volume were compared using an analysis of covariance (ANCOVA) adjusting for the covariates of age, gender, BMI, baseline cartilage defect grade, and baseline patella bone volume. To explore between group differences in cartilage defect changes (i.e., progression and regression), Chi-squared and Fisher’s exact tests were used. If significant main effects were identified, post hoc comparisons were performed using Fisher’s least significant difference (LSD). Univariate and multivariate linear regression was used to examine the relationship between baseline cartilage defects and cartilage volume annual percentage change in all ACLR participants before and after adjusting for age, gender, BMI, baseline patella bone volume, and presence of meniscal pathology. All statistical analyses were performed in SPSS (IBM, Chicago, USA) version 24.0 with significance set to *p* < 0.05.

## Results

As per our previous study [23], 66 participants returned for follow-up assessment (32 ACLR isolated, 25 ACLR combined, and 9 controls) and their characteristics are shown in Table 1. Between-group differences have been outlined previously [23] and included i) a higher BMI for the ACLR combined group compared to the ACLR isolated group (*p* = 0.007) and, ii) longer follow-up time interval between MRI assessments for the control group compared to both ACLR groups (*p* < 0.001). Reasons for drop-out have also been previously reported [23] and there was no significant difference in the characteristics of ACLR and control participants of those who completed follow-up assessment and those who did not (Supplementary File Table A1; Table A2).

**Table 1:** Characteristics of participants

	ACLR isolated (***n*** = 32)	ACLR combined (***n*** = 25)	Controls (***n*** = 9)	***p*** value
Age (yrs)	30.7 (± 6.4)	30.6 (± 7.1)	28.3 (± 4.0)	0.58
Male, n (%)	19 (59)	18 (72)	8 (89)	0.24
BMI (kg/m^2^)	24.4 (± 3.2)^1^	27.0 (± 3.6) ^1^	24.6 (± 3.8)	0.02*
Time from surgery to baseline (yrs)	2.5 (± 0.4)	2.5 (± 0.4)	Not applicable	0.92
Time from baseline to follow-up MRI (yrs)	2.1 (± 0.2) ^2^	2.0 (± 0.2) ^3^	2.9 (± 0.4) ^2 3^	< 0.001*

Parametric data are presented as mean (± standard deviation)

*ACLR* anterior cruciate ligament reconstruction, *BMI* body mass index, *MRI* magenetic resonance imaging

*Significant difference (*p* < 0.05). Post hoc was significantly different for ^1^ isolated ACLR versus combined ACLR, ^2^ isolated ACLR versus controls, ^3^ combined ACLR group versus controls

### Within-group comparisons

#### Cartilage volume

As shown in Table 2, mean patellar cartilage volume increased in both the ACLR isolated group [mean change (95% confidence interval, CI) 220 (139, 301) mm^3^] and the ACLR combined group [126 (26, 226) mm^3^] while the control group showed no significant change over follow up period [9 (− 165, 183) mm^3^].

**Table 2:** Baseline and follow-up patellar cartilage volume, cartilage volume change, and adjusted annual percentage change

	ACLR isolated (***n*** = 32)	ACLR combined (***n*** = 25)	Controls (***n*** = 9)	***p*** value
Baseline cartilage volume (mm^3^)	3328 (822)	3470 (555)	3830 (836)	0.20
Follow-up cartilage volume (mm^3^)	3548 (854)	3596 (634)	3839 (937)	0.62
Patellar cartilage volume change (mm^3^)	220 (139, 301)^1 a^	126 (26, 226) ^a^	9 (−165, 183)^1^	0.046*
Annual percentage change in patellar cartilage volume (%)^**#**^	3.5 (2.3, 4.8)^1 2^	1.3 (−0.1, 2.8)^2^	−0.1 (−2.5, 2.3)^1^	0.01*

Baseline and follow-up patellar cartilage volume were presented as mean (SD). Patellar cartilage volume change and annual percentage change in patellar cartilage volume are presented as mean (95% confidence interval). Patellar cartilage volume change = follow-up volume - baseline volume, thus positive values represent an increase in cartilage volume

*Significant difference (*p* < 0.05). ^#^Adjusted for age, gender, BMI, baseline cartilage defect score, and baseline patellar bone volume. Post hoc was significantly different for ^1^ isolated ACLR versus controls, ^2^ isolated ACLR versus combined ACLR, ^a^ follow-up versus baseline

#### Cartilage defects

ACLR isolated group showed a decrease in patellar cartilage defect scores (*p* = 0.02; Z = − 2.33; *r* = 0.3; Supplementary File Table A3).

### Between-group comparisons

#### Cartilage volume

**Fig. 2:**
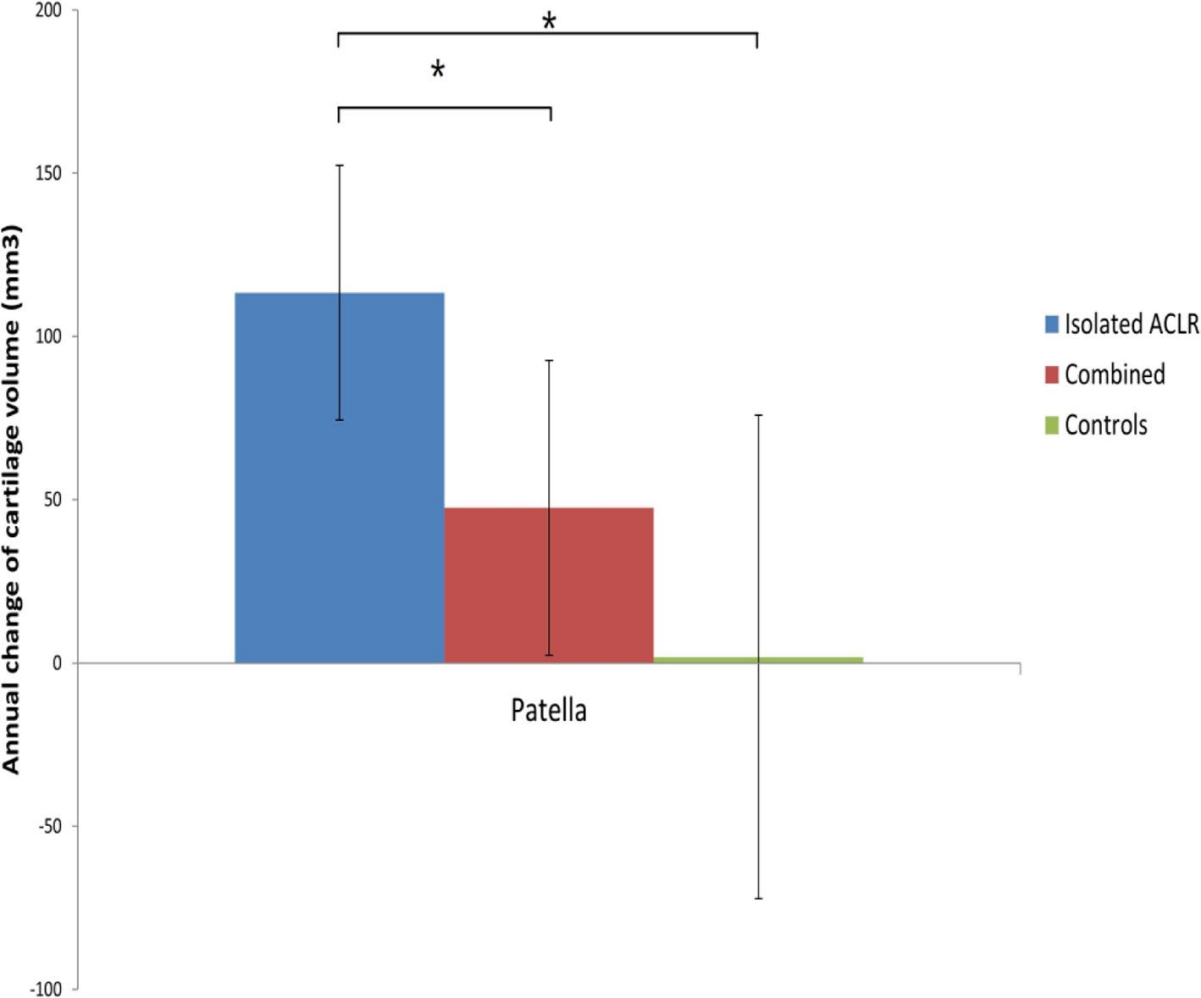
Adjusted annual changes of cartilage volume in the three groups. * Significant difference (*p* < 0.05)

The ACLR isolated group had greater annual percentage change in cartilage volume than the control group (*p* = 0.008, mean difference 3.6, 95% CI 1.0, 6.3%) and ACLR combined group (*p* = 0.028, mean difference 2.2, 95% CI 0.2, 4.2%). However, no significant differences were found between the ACLR combined and control groups (Table 2**).** The same finding was observed in the adjusted annual changes in patellar cartilage volume (Fig. 2 Adjusted annual changes of cartilage volume in the three groups. * Significant difference (*p* < 0.05)).

#### Cartilage defects

Most participants in each group exhibited stable cartilage defects (Table 3). Although ACLR groups showed cartilage defect regression in a quarter of participants (25% of ACLR isolated and 24% of ACLR combined), no significant difference was found.

**Table 3:** Patellar cartilage defects change in each group given as number (%)

	ACLR isolated (***n*** = 32)	ACLR combined (***n*** = 25)	Controls (***n*** = 9)	***p*** value
*Baseline defect score*				0.26
Grade 0	18 (56%)	15 (60%)	7 (78%)	
Grade 1	12 (38%)	7 (28%)	1 (11%)	
Grade 2	1 (3%)	3 (12%)	0 (0)	
Grade 3	1 (3%)	0 (0)	0 (0)	
Grade 4	0 (0)	0 (0)	1 (11%)	
*Follow-up defect score*				0.42
Grade 0	25 (78%)	20 (80%)	7 (78%)	
Grade 1	5 (16%)	2 (8%)	1 (11%)	
Grade 2	1 (3%)	3 (12%)	0 (0)	
Grade 3	1 (3%)	0 (0)	0 (0)	
Grade 4	0 (0)	0 (0)	1 (11%)	
*Defect changes*				
Progression	1 (3%)	2 (8%)	0 (0)	0.73
Stable	23 (72%)	17 (68%)	9 (100%)	0.18
Regression	8 (25%)	6 (24%)	0 (0)	0.29

### Associations between baseline patellar cartilage defects and annual percentage change in patellar cartilage volume amongst ACLR groups

Among ACLR participants, baseline patellar cartilage defect score was positively associated with annual percentage patellar cartilage volume change in univariate analysis (regression coefficient (B) = 0.013; 95% CI 0.001, 0.026; *p* = 0.04). The significant association remained after adjustment for age, gender, BMI, baseline patella bone volume, and presence of meniscal pathology, where higher baseline patella cartilage defect score was associated with an increase in patellar cartilage volume over 2 years (B = 0.014; 95% CI 0.001, 0.027; *p* = 0.03).

## Discussion

This longitudinal study examined changes in patellar cartilage morphologic features (i.e., cartilage volume and cartilage defects) in ACLR knees with or without combined meniscal pathology from 2.5 to 4.5 years post-surgery, as well as a control group assessed over 2 years. ACLR groups demonstrated an increase in patella cartilage volume whilst control participants exhibited no change over the study period. Moreover, the isolated ACLR group showed a higher level of patella cartilage volume increase than the combined ACLR and control groups. Patellar cartilage defect scores significantly regressed in the isolated ACLR group. Finally, in ACLR participants, a positive association was identified between baseline patellar cartilage defect scores and the increase in patellar cartilage volume over the 2-year study period.

Consistent with H_1_, both isolated and combined ACLR knees showed significant increases in patellar cartilage volume at follow-up compared to baseline, while the patellar cartilage volume of the control group exhibited no significant change. Increase in patellar cartilage volume in ACLR knees is consistent with results of several recent longitudinal studies that have also reported increased knee cartilage volume or thickness within 1–5 years post-ACLR [15–17, 23] - albeit in the TF joint compartments. These findings may be indicative of early OA development that where cartilage increases have been reported prior to cartilage loss and may be due to tissue hypertrophy, repair and swelling [15]. In contrast to the current study, Culvenor et al. [14] reported decreased PF cartilage thickness at 5-years following ACLR- a finding which, given the follow-up period, may be reflective of a more advanced stage of cartilage degeneration than the current study. In the early stages of cartilage degeneration, cartilage increase is suggestive of accelerated metabolism and increased water - an adaptive response to repair cartilage damage and withstanding mechanical load [27, 28]. Whilst identifying the biomechanical mechanisms contributing to patella cartilage increase are beyond the scope of this study, PFJ ‘*underloading*’ during running in a comparable cohort of ACLR patients has been reported [29]. Decreased PFJ loading has been associated with early degenerative changes in ACL-transected animals [30, 31]. In humans, a relationship between TF joint underloading and the development of early-onset TF osteoarthritis has been identified [32]. Clearly, the definitive biomechanical conditions that contribute to the pathogenesis of PFJ OA post-ACLR need to be the focus of future studies.

In contrast to H_2_, ACLR isolated knees exhibited a greater increase in annual percentage change in patella cartilage volume than the combined ACLR and control knees. Meniscectomy and meniscal injuries have been associated with a higher prevalence of TF and PF joint OA following ACLR [7, 33, 34]. For this reason, we hypothesised (H_2_) that combined ACLR knees would demonstrate more pronounced cartilage volume change compared to isolated ACLR and control knees. However, our results indicate the opposite, and no differences were found between combined ACLR and control knees. This unexpected finding may be related to the fact that degenerative changes occur across cartilage sub-regions at different rates. In this respect, Eckstein et al. [15] whilst adopting a different technique for quantifying cartilage morphology, reported concurrent TF cartilage thickening and thinning in different sub-regions within the same cartilage compartment in post-ACLR participants. It has been widely accepted that an increase in cartilage volume precedes cartilage thinning during the process of cartilage degeneration [28]. Overall cartilage morphology is a direct result of the balance between cartilage hypertrophy and loss.

The isolated ACLR group experienced greater patellar cartilage volume increase suggesting that, on balance, increasing cartilage volume was the predominant change across the plate sub-regions. In contrast, combined ACLR knees may have been undergoing a higher level of cartilage thinning in some sub-regions due to more rapid degeneration compared with isolated ACLR knees. This argument is also supported in the TF joint, as our previous research has demonstrated the same between-group difference in the lateral tibia [23].

Contrary to H_1_, the majority of participants in each of the three groups had stable cartilage defects meaning defect grades were unchanged between baseline and follow up assessment time points. The stability of the cartilage defects in both ACLR groups suggests that the cartilage defects persist from 2.5–4.5 years post-ACLR. This notion is supported by Potter et al. (2012) who found cartilage defects in all 40 patients at the time of ACL injury, and minimal subsequent change in cartilage defect size until ~ 7 years post injury, at which point there was a marked increase in defect [20]. It is likely that the increase at this latter time represents acceleration of the degenerative processes, and is consistent with the higher rates of OA development observed around this time period (i.e., over 10 years) post-injury [2, 4].

Notably and contrary to H_1_, patellar cartilage defect scores significantly regressed in the isolated ACLR group and 25% of knees exhibited improvements in PF cartilage defects from baseline to follow-up. These improvements seem to be independent of concurrent meniscal injury, as 24% of the combined ACLR knees also showed cartilage defect improvement. This finding is different from previous studies showing one-way progression of patellar cartilage defects from 2 to 11 years following ACLR [20, 35]. In support however, another study [36] demonstrated that in a relatively young (i.e., mean age of 45 years) cohort of 325 participants, largely without radiographic OA, 13% of participants showed improvement in patellar cartilage defects over two-year period [36]. Improvement in cartilage defects, due to cartilage synthesis or swelling, reflects an attempt to repair cartilage damage and withstand mechanical load [28]. The natural history of cartilage defects was also age-related. In older groups, improvement of cartilage defects appears to be less common. Specifically, in a study of 395 participants with mean age 62.7 years, 26% cartilage defects progressed at the patella over 3 years, with the majority of defects remaining stable, and defect improvement rarely occurring (~ 1% of participants) [37]. In another study of 86 healthy participants with a mean age of 57 years, approximately 36% had worsening in patellar cartilage defects, while approximately 18% improved over 2 years [38]. Moreover, a recent study reported that 17% of ACLR participants had cartilage defects or osteophytes in the PF joint and, as such, were categorised as exhibiting MRI-defined PF joint OA [6]. Although defining early OA is of great value [39], results of the current study suggest that the definition of early OA should be carefully selected. Specifically, using the presence of mild cartilage defects as a diagnostic criterion for early OA may be inappropriate in either research or clinical setting, considering the regression of cartilage defects in a substantial portion of ACLR patients.

Contrary to H_2_, there were no differences in changes in cartilage defects between the three groups. This lack of difference between groups may be attributable to the small change in cartilage defects over the 2-years, and/or the relatively small sample size with a lack of power to detect statistical differences.

In support of H_3_, higher baseline cartilage defect scores at the patella of ACLR participants were associated with greater patellar cartilage volume increases over the following 2 years. The positive relationship indicates that mildly disturbed cartilage homeostasis (i.e., ICRS 1–2 cartilage defects) was associated with cartilage adaptation, which may be indicative of early cartilage degeneration. The positive association between cartilage defects and increases in cartilage volume is consistent with our previous finding in the lateral tibia [22]. It is important to note that ACLR participants in this study were different from those included in previous studies which reported that more severe cartilage defects (i.e., ICRS 3–4) were associated with an increased likelihood of developing radiographic OA [33] and worse patient-report outcomes [40–43].

This study has several strengths. Firstly, this is the first study to compare the change in patellar cartilage morphology between ACLR participants with and without concomitant meniscal pathology. Secondly, in contrast to most other longitudinal studies, we included an age-matched control group for comparative purposes. By contrast, our study also has several limitations. Firstly, as outlined in our previous study [23], around half of the participants were lost to follow-up. Importantly, no difference in participant characteristics were identified between those participants who remained in the study and those lost to follow-up. Secondly, although gender was included as a covariate in our statistical comparisons, there was an unequal distribution of males and females in the three groups. Thirdly, the sample size was relatively small for the combined ACLR group and the control group, which could reduce the statistical power of the study [23].

## Conclusions

Patellar cartilage hypertrophy was evident in ACLR participants with and without concomitant meniscal pathology at ~ 4.5 years post-surgery. However, it may be inferred that patellar cartilage degenerative changes occur at different rates given the more pronounced increase in patellar cartilage volume in the ACLR isolated group compared to the ACLR combined group. Cartilage defects were stable in the majority of ACLR participants; however, the severity of patellar cartilage defects at baseline influenced the cartilage hypertrophic response over the subsequent ~ 2 years. Future studies should investigate the relationship between patellar cartilage morphology and gait-related patellofemoral contact forces in the early years following ACLR.

## Supplementary Information

**Additional file 1 **: **Table A1**. Characteristics of ACLR participants who completed the study and those who were lost to follow-up. **Table A2**. Characteristics of control participants who completed the study and those who were lost to follow-up.

**Additional file 2 **: **Table A3.** Median (IQR) baseline and follow-up patellar cartilage defect score with pre-post Wilcoxon test in each group.

## Data Availability

The datasets used and analysed in the current study are available from the corresponding author on reasonable request.

## Abbreviations

ACL: Anterior cruciate ligament
ACLR: Anterior cruciate ligament reconstruction
ANCOVA: Analysis of covariance
BMI: Body mass index
CI: Confidence Interval
ICCs: Intra-class correlation coefficients
ICRS: International Cartilage Repair Society
IUD: Intrauterine device
LSD: Least significant difference
MRI: Magnetic resonance imaging
OA: Osteoarthritis
PF: Patellofemoral
TF: Tibiofemoral
