# Supplementary material for: Patellar cartilage increase following ACL reconstruction with and without meniscal pathology: a two-year prospective MRI morphological study
Source: BMC Musculoskelet Disord. 2021 Oct 28;22:909. doi: 10.1186/s12891-021-04794-5 (PMC8555213; doi:10.1186/s12891-021-04794-5)
Supplement: Supplementary file 2 — Additional file 2 : Table A3. Median (IQR) baseline and follow-up patellar cartilage defect score with pre-post Wilcoxon test in each group. [file 12891_2021_4794_MOESM2_ESM.docx]

Table A3 Median (IQR) baseline and follow-up patellar cartilage defect score with pre-post Wilcoxon test in each group

| **Group** | **Baseline** | **Follow-up** | ***p* value** |
| --- | --- | --- | --- |
| **ACLR isolated (n = 32)** | 0 (0, 1) | 0 (0, 0) | 0.02* |
| **ACLR combined (n = 25)** | 0 (0, 1) | 0 (0, 0) | 0.13 |
| **Controls (n = 9)** | 0 (0, 1) | 0 (0, 1) | 1.0 |

* Significant difference (*p* < 0.05).
